# Environmental manipulations generate bidirectional shifts in both behavior and gene regulation in a crossbred mouse model of extremes in trait anxiety

**DOI:** 10.3389/fnbeh.2014.00087

**Published:** 2014-03-18

**Authors:** Natalia Yurievna Chekmareva, Sergey V. Sotnikov, Rebekka P. Diepold, Roshan R. Naik, Rainer Landgraf, Ludwig Czibere

**Affiliations:** ^1^Department of Behavioral Neuroendocrinology, Max Planck Institute of PsychiatryMunich, Germany; ^2^Department of Normal Physiology, Sechenov First Moscow State Medical UniversityMoscow, Russia

**Keywords:** anxiety-related behavior, gene expression, allele-specificity, *Crhr1. Hmgn3*, enriched environment, chronic mild stress

## Abstract

Although gene-environment interactions are known to significantly influence psychopathology-related disease states, only few animal models cover both the genetic background and environmental manipulations. Therefore, we have taken advantage of the bidirectionally inbred high (HAB) and low (LAB) anxiety-related behavior mouse lines to generate HAB × LAB F1 hybrids that intrinsically carry both lines’ genetic characteristics, and subsequently raised them in three different environments—standard, enriched (EE) and chronic mild stress (CMS). Assessing genetic correlates of trait anxiety, we focused on two genes already known to play a role in HAB *vs.* LAB mice, corticotropin releasing hormone receptor type 1 (*Crhr1*) and high mobility group nucleosomal binding domain 3 (*Hmgn3*). While EE F1 mice showed decreased anxiety-related and increased explorative behaviors compared to controls, CMS sparked effects in the opposite direction. However, environmental treatments affected the expression of the two genes in distinct ways. Thus, while expression ratios of *Hmgn3* between the HAB- and LAB-specific alleles remained equal, total expression resembled the one observed in HAB *vs*. LAB mice, i.e., decreased after EE and increased after CMS treatment. On the other hand, while total expression of *Crhr1* remained unchanged between the groups, the relative expression of HAB- and LAB-specific alleles showed a clear effect following the environmental modifications. Thus, the environmentally driven bidirectional shift of trait anxiety in this F1 model strongly correlated with *Hmgn3* expression, irrespective of allele-specific expression patterns that retained the proportions of basic differential HAB *vs.* LAB expression, making this gene a match for environment-induced modifications. An involvement of *Crhr1* in the bidirectional behavioral shift could, however, rather be due to different effects of the HAB- and LAB-specific alleles described here. Both candidate genes therefore deserve attention in the complex regulation of anxiety-related phenotypes including environment-mediated effects.

## Introduction

The genetic basis for phenotypic variation is provided by both sequence-based polymorphisms and epigenetic regulation. To address the latter, intra- and inter-strain differences in behavior are the best known examples of gene-environmental interactions to study in mice (Hovatta et al., 2005; Alter et al., 2008). Applying a wide variety of breeding, embryo transfer and cross-fostering approaches, significant breakthroughs have been accomplished by demonstrating the importance of *in utero* and postnatal environments and parent-of-origin effects on individual behavior (Rhees et al., 1999; Francis et al., 2003; Bartolomucci et al., 2004; Kalueff et al., 2007).

To implement these phenotypic changes, alterations in synaptic plasticity or total gene expression (tGEx) can be a driving force (Hovatta and Barlow, 2008), but recent literature also suggests that allele-specific gene expression (asGEx), especially as in genetic imprinting, can exert a strong impact on the developing phenotype including the predisposition or development of pathologic states (Walston et al., 2009; Gregg et al., 2010). At the same time, asGEx of non-imprinted genes is also a commonly known phenomenon (Cowles et al., 2002; Lo et al., 2003; Yan and Zhou, 2003).

Focusing on the genetic basis of anxiety-related behavior, high (HAB) and low (LAB) anxiety-related behavior mice were selectively inbred starting with outbred CD-1 mice. Therefore, for each generation of breeding, mice were tested on the elevated plus-maze (EPM) to select for the most and least anxious individuals as reflected by the time spent on the open arms (Krömer et al., 2005; Sartori et al., 2011). Breeding and phenotypic characterization of HAB and LAB mice for more than 45 generations provided the basis for an extreme phenotypic divergence in a variety of behavioral paradigms reflecting not only anxiety-related behavior (EPM, light-dark box (LDB)) but also depression-like behavior as indicated by forced swim (FST) or tail suspension tests (TST; Bunck et al., 2009; Yen et al., 2013). The stability of behavioral characteristics of these lines allowed for exploring the genetics behind these phenotypes that led to the identification of some candidate genes of anxiety including cathepsin B (Czibere et al., 2011), arginine vasopressin (Kessler et al., 2007; Bunck et al., 2009), transmembrane protein 132d (Erhardt et al., 2011), glyoxalase 1 (Krömer et al., 2005), corticotropin releasing hormone receptor type 1 (*Crhr1*; Sotnikov et al., 2014) and high mobility group nucleosomal binding domain 3 (*Hmgn3*; Czibere et al., 2011).

As HAB and LAB animals represent the poles of an anxiety continuum (Landgraf et al., 2007), we generated HAB × LAB F1 hybrids that intrinsically carry both lines’ genetic characteristics. These hybrids were exposed to two different environmental treatments: a more pleasant, beneficial—enriched environment (EE) and an unpleasant, debilitating—chronic mild stress (CMS) one. tGEx and asGEx patterns of *Crhr1* and *Hmgn3* were assessed after the environmental modifications as candidate genes known to consistently differ in expression and bear polymorphisms in HAB *vs.* LAB mice (Czibere et al., 2011; Sotnikov et al., 2014). For gene expression analyses, we focused on the basolateral amygdala (BLA), since in this brain region bidirectional shifts in *Crhr1* expression were observed upon environmental modifications in HAB and LAB mice after EE and CMS exposure, respectively (Sotnikov et al., 2014). Moreover, using *in situ* hybridization, we recently compared *Crhr1* mRNA expression between standard-housed and EE HAB mice in different brain structures (including the prefrontal and cingulate cortices, hippocampus, dentate gyrus, amygdala and PVN) (Sotnikov et al., submitted). These data clearly point to the amygdala as an exclusive brain region, where *Crhr1* is affected by environmental manipulation. Furthermore, our electrophysiological measurements (Avrabos et al., 2013), *c-fos* expression data (Sotnikov et al., submitted) and the detailed analysis of *Crhr1* expression comparing standard-housed and EE mice in different amygdalar subnuclei further support the idea that, although environmental manipulation partially affected gene expression in the lateral and medial subdivisions, the major differences were observed in the basolateral and central parts. In addition, Van Pett et al. (2000) and Kühne et al. (2012) reported no or very low levels of *Crhr1* expression in the central amygdala.

In the current study, we show that both environmental modifications, EE and CMS, are valid paradigms to induce a bidirectional shift of behavior in HAB × LAB F1 mice. This change in trait anxiety is accompanied by a corresponding asGEx of *Crhr1* in the BLA, although not affecting tGEx, and by a change of tGEx for *Hmgn3*, where the changes are in line with the expected tGEx patterns derived from the parental HAB and LAB lines, respectively.

## Materials and methods

### Animals

All animal studies were conducted in accordance with the Guidelines for the Care and Use of Laboratory Animals and the approval of the Government of Upper Bavaria. 10 HAB and 10 LAB mice were used for generating F1 hybrids. To minimize potential epigenetic influences of different maternal behavior of HAB or LAB females (Kessler et al., 2011) on their offspring’s phenotype, only ♀ HAB × ♂ LAB crossmates were used in the experiment. To exclude effects of the estrous cycle, only male mice were used in the experiment. On postnatal day (PND) 15, pups were randomly distributed to form the following experimental groups: control (*n* = 15), EE (*n* = 16), CMS (*n* = 20). All animals were weaned on PND 28 and kept in a controlled environment in groups of 3 per cage with stable temperature (22.5 ± 1°C), relative air humidity (45 ± 5%) and with a 12/12 h light/dark cycle with lights on at 8 a.m. Food and water were provided *ad libitum*. EE and CMS groups were treated according to the protocols below.

### Enriched environment (EE)

The EE paradigm was used as described before (Avrabos et al., 2013) and included bigger home cages (Makrolon cage type IV; 55 × 30 × 20 cm), filled with additional nesting material and supplied with 6 ± 0.5 cm thick layer of wood chips (LIE E—001, Abedd Lab and VET Service, Vienna, Austria), a plastic inset (22 × 16 × 8 cm) and tunnel (19.5 × 6 × 6 cm), a wooden ladder and scaffold to offer climbing structures. During the weekly change, half of the nesting material was transferred into the fresh cage. The EE paradigm comprised two 14-days periods, called partial and full enrichment. During partial enrichment (PND 15–28), all litters with dams were transferred to EE for 6 h per day. On PND 28, pups were weaned and grouped by three for full EE until PND 42. Behavioral phenotyping was conducted in the order EPM, LDB, TST and FST with 48 h test intervals, 24 h of rest was provided before behavioral testing.

### Chronic mild stress (CMS)

CMS was applied in parallel to EE (Avrabos et al., 2013). The stress procedure did not include more than 2 stressors per day. Mice were subjected to different kinds of stressors, including maternal separation (PND 15–28, 3 h per day), restraint stress (PND 28–42, 30 min per day), cage tilting (3 times 45° for 7 h), damp sawdust (twice overnight), placement to an empty cage (3 times overnight), placement to an empty cage with water at the bottom (twice for 1 h), inversion of the light/dark cycle (3 times), overcrowding (twice overnight) or paired housing (once overnight), white noise (85 dB 3 times for 3 h), stroboscopic light (3 times for 7 h), foot shocks (once 0.7 mA with 3 s duration). Behavioral phenotyping was conducted in the order described for EE.

### Elevated plus-maze (EPM)

The plus-shaped EPM was made out of dark gray PVC and consisted of two opposing open (30 × 5 cm, with light intensity changing gradually from 300 to 50 lx) and two opposing closed arms (30 × 5 × 15 cm, with light intensity 10 lx) connected by a central platform (5 × 5 cm). The EPM was located 40 cm above the floor. At the beginning of each 5-min trial, the mouse was placed on the central platform facing a closed arm. Before each test session, the apparatus was cleaned with water containing a detergent. Behavior was monitored by a video camera fixed above the EPM. The animals’ behavior was scored automatically by software (Any-Maze v. 4.82, Stoelting, Wood Dale, IL, USA). Adequate software tracking was controlled continuously by an experienced observer. The following parameters were assessed: distance traveled, percentage (%) of time spent on the open arms, latency to the first open arm entry and the number of entries to the open arms.

### Light-dark box (LDB)

The LDB was a two-chambered apparatus, open at the top, with a smaller black-colored, dark compartment (15 × 20 × 26 cm; 15 lx) and a white-colored, brightly lit bigger one (29 × 20 × 26 cm; 400 lx). Mice started each 5-min trial in the dark compartment; their behavior was scored by the Any-Maze software. Parameters assessed in this test were: % time spent and distance traveled in the light compartment, the latency to enter the light compartment, vertical rearings and total distance traveled. The apparatus was cleaned with water containing a detergent before each test session.

### Tail suspension test (TST)

Mice were suspended by the end of their tail to a bar 35 cm above the floor for 6 min, their behavior was videotaped, and the duration of total immobility scored by a trained observer blind to line or treatment using Eventlog 1.0 software (EMCO Software, Reykjavik, Iceland).

### Forced swim test (FST)

Animals were placed into a glass cylinder (11 cm in diameter) filled with 1600 ml water of 23°C for 6 min and videotaped. Struggling (forepaws brake through the water surface) and floating (the animal is immobile) were scored by a trained observer blind to line or treatment using Eventlog 1.0.

### Corticosterone radioimmunoassay (RIA) and brain tissue preparation

Mice were sacrificed between 9 and 12 a.m., 48 h after the last behavioral test. Animals were deeply anesthetized with Forene and subsequently decapitated. Trunk blood was collected and centrifuged at 4000 rpm for 10 min at 4°C to separate plasma from the cellular content. 10 μl of 1:13.5 diluted plasma was used to determine the concentration of corticosterone using a radioimmunoassay (RIA) kit (DRG Diagnostica, Marburg, Germany) according to the manufacturer’s instructions. Intra- and inter-assay coefficients were below 10%.

Brains were taken, snap-frozen, cut into 200 μm slices mounted to Superfrost microscope slides (Menzel, Braunschweig, Germany) in a cryostat (Microm MH50, Microm, Walldorf, Germany). The BLA was sampled from frozen slices applying a Ø 0.5 mm sample corer (Fine Science Tools, Heidelberg, Germany) according to the coordinates described previously (Czibere et al., 2011).

### RNA extraction, reverse transcription and total gene expression (tGEx)

Total RNA was extracted from the BLA tissue punches using a Trizol (Invitrogen, Karlsruhe, Germany) chloroform protocol (Czibere et al., 2011). RNA concentration was assessed on a NanoPhotometer (Implen, Munich, Germany). Approximately 100 ng of total RNA was used for cDNA conversion. Reverse transcription was performed according to the protocol of the High-Capacity cDNA reverse transcription kit (Applied Biosystems, Foster City, CA) using random primers. tGEx was measured by quantitative PCR (qPCR) using QuantiFast SYBR Green PCR Kit (Qiagen, Hilden, Germany) on a LightCycler 2.0 instrument (Roche Diagnostics, Mannheim, Germany) with the following primers (Sigma Aldrich, Taufkirchen, Germany): *Crhr1* forward: GCC CCA TGA TCC TGG TCC TGC and reverse: CCA TCG CCG CCA CCT CTT CC and *Hmgn3* forward: AGG TGC TAA GGG GAA GAA GG and reverse: GTC CCG AGA GGT ACG TGA AA. Analysis was performed using the comparative *C_t_* method. All samples were analyzed in duplicates and normalized to the housekeeping genes *Polr2b* forward: CAA GAC AAG GAT CAT ATC TGA TGG, reverse: AGA GTT TAG ACG ACG CAG GTG and *B2mg* forward: CTA TAT CCT GGC TCA CAC TG and reverse: CAT CAT GAT GCT TGA TCA CA, respectively.

### Quantitative analysis of allele-specific expression (asGEx)

Single-nucleotide polymorphisms (SNP) in the coding sequences of the analyzed genes were used for designing allele-specific primers for a qPCR assay. Namely, SNPs rs27025657 A/G in *Crhr1*, rs30291581 A/G and rs30360110 G/A in *Hmgn3* genes were identified as differing consistently between the HAB and LAB lines (Brenndörfer, personal communication; Czibere et al., 2011). The primers for amplification carried line-specific nucleotides (displayed in italic) with the 3′ penultimate nucleotide altered (mismatched, displayed underlined) to increase specific binding at the 3′ end. Allele-specific primers *Crhr1*: LAB-specific forward: AAG AGG TGG CGG CT*G*, HAB-specific forward: AAG AGG TGG CGG CT*A* and common reverse GAT GGG AAG GCT GCC; *Hmgn3*: LAB-specific forward: ATG CAC ACG GGA GCG C*G*, HAB-specific forward: ATG CAC ACG GGA GCG C*A* and LAB-specific reverse AG ACA AGG CAG GAA GGC CTT A*T*, HAB-specific reverse: AG ACA AGG CAG GAA GGC CTT A*C.* The ability of primers to amplify only line-specific products was evaluated in an additional setup, where different proportions of HAB and LAB cDNA were mixed (9:1/1:1/1:9) and used for quantitative analysis with sequence-specific primers. Results were calculated relative to one primer and plotted as a standard curve (Figures 2A, B). Higher expression of the specific product with its corresponding primer and lower with the non-matching primer indicated sequence-specific amplification. cDNA of F1 animals from the BLA was used for qPCR with each set of primers. Analysis was performed based on the standard curves. Results are presented as relative percentage of tGEx within each treatment group.

**Figure 1:**
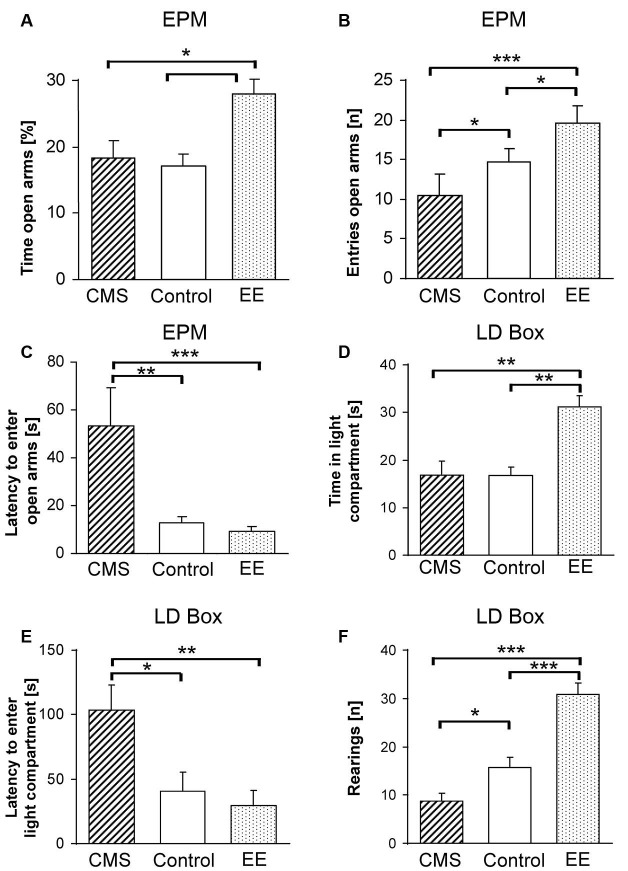
**Phenotypic measures of F1 mice reflecting changes in anxiety-related behavior upon environmental modifications.** Phenotypes were assessed on the elevated plus-maze (EPM) with **(A)** percent time spent on the open arms, **(B)** number of entries to the open arms, **(C)** latency to the first entry to an open arm and in the light-dark box (LDB) with **(D)** time spent in the light compartment, **(E)** latency to the first entry to the light compartment and **(F)** number of rearings after 4 weeks of chronic mild stress (CMS; dashed bars) or enriched environment (EE; dotted bars). Data are shown as means + SEM; * *p* < 0.05; ** *p* < 0.01; *** *p* < 0.001.

**Figure 2:**
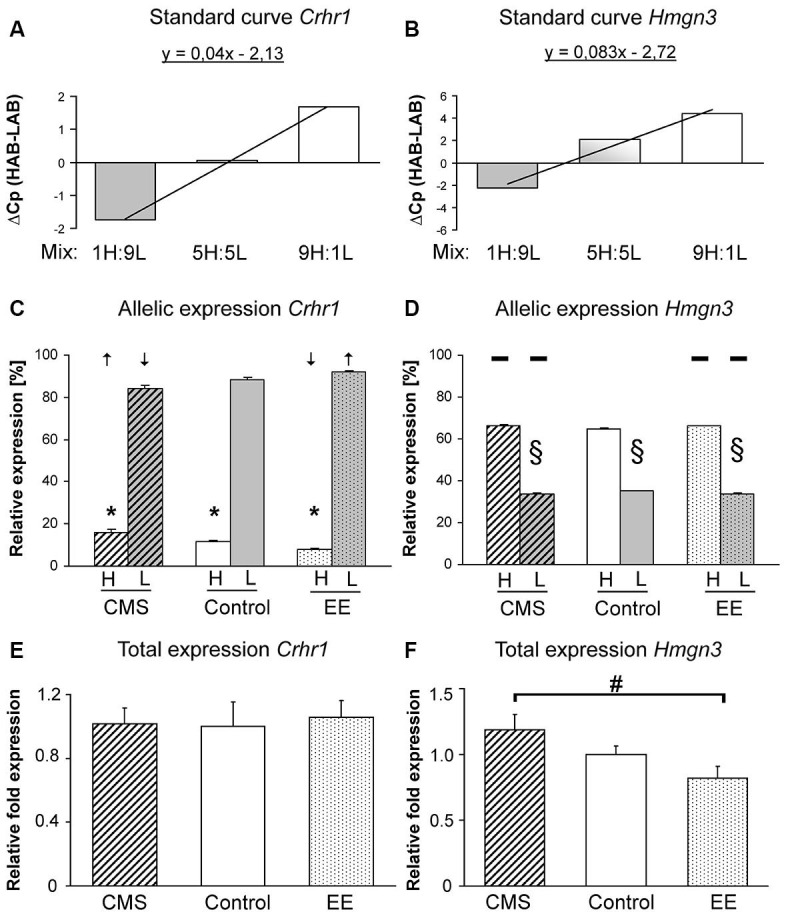
**Expression assays and patterns for *Crhr1* and *Hmgn3***. Assay development for HAB and LAB-specific expression analyses providing the proof for specific amplification of *Crhr1* **(A)** and *Hmgn3* **(B)**. Allele-specific expression of *Crhr1* **(C)** and *Hmgn3* **(D)** with arrows indicating shifts in allele-specific expression after enriched environment (EE) or chronic mild stress (CMS). Total gene expression of *Crhr1* **(E)** and *Hmgn3* **(F)** between EE and CMS F1 mice. Data are shown as means + SEM; * *p* < 0.01 for *Crhr1* HAB allele-specific expression compared to the LAB allele, § *p* < 0.01 for *Hmgn3* LAB allele-specific expression compared to the HAB allele, # *p* < 0.05 for *Hmgn3* total expression in CMS *vs*. EE groups.

### Data analysis

Statistical analysis was performed using PASW Statistics 18 (SPSS, Quarry Bay, Hong Kong). The analysis of behavior after treatment (CMS, control and EE) was done by one-way analysis of variance (one-way ANOVA; factor: treatment). Bonferroni *post-hoc* test was used to correct for multiple testing. Overall differences in asGEx were tested using Kruskal-Wallis test followed by Mann-Whitney *U*-tests for 2-group comparisons. All results were considered statistically significant at *p* < 0.05.

## Results

### Elevated plus-maze (EPM)

No effect of environmental treatment on locomotor activity as measured by the total distance traveled was found between control, EE and CMS groups (*F*_(2,48)_ = 0.55, *p* < 0.57). However, significant differences were observed in % time spent on the open arms (*F*_(2,48)_ = 4.86, *p* < 0.01), latency to enter the open arm (*F*_(2,48)_ = 5.40, *p* < 0.007) and entries to the open arms (*F*_(2,48)_ = 11.30, *p* < 0.0001). Bonferroni *post-hoc* tests showed a significant treatment effect between groups with EE mice having higher % time spent on the open arms in comparison to control (*p* < 0.03) and CMS (*p* < 0.02) mice (Figure 1A), the CMS treated group displayed a higher latency to enter the open arms and a higher number of entries to the open arms compared to controls (*p* < 0.03 and *p* < 0.04) and EE (*p* < 0.01 and *p* < 0.0001), respectively (Figures 1B, C). A significant difference in the number of entries to the open arms was observed between control and EE groups (*p* < 0.05, Figure 1B).

### Light-dark box (LDB)

One-way ANOVA indicated a significant effect of treatment on the % of time spent in the light compartment (*F*_(2,48)_ = 10.10, *p* < 0.0002), where the EE group spent significantly more time in comparison to controls (*p* < 0.0001) and CMS (*p* < 0.002, Figure 1D). Moreover, the CMS treated group showed a significantly higher latency to enter the light compartment compared to control and EE mice (*F*_(2,48)_ = 6.57, *p* < 0.003; *p* < 0.02 *vs*. control and *p* < 0.005 *vs.* EE, Figure 1E). A difference in explorative behavior, indicated by the number of rearings, was observed between all experimental groups (*F*_(2,48)_ = 34.05, *p* < 0.0001), with the CMS group having lower numbers in comparison to the control (*p* < 0.04) group, and EE exhibiting the highest numbers (*p* < 0.0001 for both control and CMS, Figure 1F). However, we observed significantly higher locomotor activity of the EE treated group (*F*_(2,48)_ = 17.53, *p* < 0.001 for both groups) and higher % distance traveled in the light compartment (*F*_(2,48)_ = 8.38, *p* < 0.003 for both groups), but due to different sizes of the dark and light chambers and a different explorative activity, these data should be interpreted with caution.

### Tail suspension test (TST), Forced swim test (FST) and blood plasma corticosterone

Both behavioral tests indicated a significant difference in passive coping strategies (time spent immobile) between the treated groups (*F*_(2,48)_ = 4.93, *p* < 0.01 and *F*_(2,48)_ = 3.37, *p* < 0.04, respectively). However, this effect was only observed in the CMS group (higher time spent immobile) in the TST (*p* < 0.04 *vs*. control and *p* < 0.06 *vs.* EE) and in FST (*p* < 0.05 with EE), with no differences between the EE and control groups. The analysis of plasma corticosterone concentrations measured at 9 a.m. revealed a group effect (*F*_(2,27)_ = 3.86, *p* < 0.03), with the CMS group showing significantly higher concentrations than the EE group (*p* < 0.04).

### Allele-specific gene expression (asGEx) and total gene expression (tGEx)

Both primer pairs of *Crhr1* and *Hmgn3* for allele-specific qPCR were able to distinguish their line-specific products in the respective reaction mixtures. Positive correlation of relative crossing points measured in defined mixtures containing increasing amounts of one specific allele indicates primer-specific products (Figures 2A, B). asGEx of HAB and LAB *Crhr1* alleles in F1 mice revealed an almost 3-fold higher expression of the LAB-specific allele (*p* < 0.001). CMS and EE exposure resulted in a regulation of asGEx with the following pattern: down-regulation of the LAB asGEx after CMS and up-regulation after EE (CMS *vs*. EE *p* < 0.002) that corresponds with an increase in HAB asGEx observed after CMS and decrease after EE (CMS *vs*. EE *p* < 0.001, Figure 2C). For *Hmgn3*, a higher asGEx was found for the HAB-specific allele (*p* < 0.002) throughout all treatment groups, however, no difference was observed after environmental manipulations (*p* < 0.11, Figure 2D)*. Crhr1* tGEx in the BLA did not differ between experimental groups (*p* < 0.89, Figure 2E), tGEx of *Hmgn3* was increased in the CMS-treated group in comparison to EE (*p* < 0.025, Figure 2F) after the environmental manipulations.

## Discussion

HAB × LAB F1 hybrids that carried both parental lines’ genetic characteristics, and underwent aversive (CMS) and beneficial (EE) environmental treatments, respectively, fulfilled our expectations based on the behavioral effects in the parental lines (Avrabos et al., 2013; Sotnikov et al., 2014). Briefly, EE mice showed decreased anxiety-related and more explorative behaviors compared to controls, while CMS promoted phenotypic changes in the opposite direction.

However, the two candidate genes analyzed, *Hmgn3* and *Crhr1*, were altered in two distinct ways, suggesting gene-specific phenomena. While expression ratios between the HAB- and LAB-specific alleles (asGEx) remained equal for *Hmgn3*, tGEx was decreased in the less and increased in the more anxious animals, i.e., EE- and CMS-exposed, just as expected from the basal tGEx levels in HAB *vs*. LAB mice (Czibere et al., 2011). On the other hand, tGEx of *Crhr1* remained unchanged between the groups—unlike in our previous observations in EE-treated HAB and CMS-exposed LAB mice (Sotnikov et al., 2014), while the relative expression of HAB- and LAB-specific alleles (asGEx) showed a clear tendency towards a treatment effect. Compared to the standard environment, the HAB-specific allele of *Crhr1* was expressed at an increased ratio after CMS and the LAB-specific allele at a higher ratio after EE, which is in line with our previous findings and, finally, with the concept that HAB-specific genetic determinants at a higher dosage confer increased anxiety-related behavior and, inversely, LAB-specific ones decreased anxiety-related behavior.

The anxiolytic effects of EE and the anxiogenic effects of CMS have been repeatedly shown in different studies (Griebel et al., 2002; Tannenbaum et al., 2002; Benaroya-Milshtein et al., 2004; Willner, 2005; Sztainberg et al., 2010). Although the treatments cannot be clearly restricted to a specific phenomenon like an effect on anxiety-related behavior, but can be accompanied by differences in brain plasticity and cognitive performance (Fares et al., 2013), we focused on anxiety-related and closely associated behaviors, as the HAB/LAB mouse model represents genetically predisposed extremes in anxiety-related behavior. Despite this rigid genetic predisposition, our previous data showed that EE and CMS can efficiently decrease anxiety in HAB mice exposed to EE and increase anxiety in LAB mice exposed to CMS (Sotnikov et al., 2014).

Here, we provide evidence that these paradigms are also applicable to HAB × LAB F1 hybrids which are heterozygous and thus carry alleles from both lines. We observed a significant reduction of anxiety in the EE-treated F1 group, indicated by higher time spent on the open arms of the EPM (Figure 1A) and an increase of time spent in the light compartment of the LDB (Figure 1D). In contrast, the CMS-treated F1 group was characterized by a higher level of anxiety-related behavior, as reflected by the lower number of entries and higher latency to enter the open arms of the EPM (Figures 1B, C) and the light compartment of the LDB (Figure 1E). Both the EE and CMS groups differed significantly in explorative activity from controls (Figure 1F). Moreover, analysis of blood samples revealed that the CMS group had higher levels of corticosterone in comparison to EE mice under basal conditions. Increased depression-like behavior was observed in the CMS group, as indicated by higher immobility times in both the FST and TST. Altogether, these data suggest that the intermediate phenotype of F1 mice can be bidirectionally shifted, i.e., the phenotypes of EE- and CMS-treated animals are appreciably and predictably different from each other and from the control group. This allowed us to study the impact of tGEx and asGEx on the phenotypic characteristics in the F1 hybrids intrinsically carrying the genetic characteristics of HAB and LAB mice. While this creates a novel mouse model by itself, since the combination of two unique genetic backgrounds results in a third one with novel options for many kinds of genetic interaction, it allows to study the extent and magnitude of involvement of single factors from both parental lines.

This model is similar to other approaches regarding the environmental modifications chosen (Griebel et al., 2002; Tannenbaum et al., 2002; Benaroya-Milshtein et al., 2004; Willner, 2005; Sztainberg et al., 2010). Other models focusing on g × e interaction either apply substances or up- or down-regulate specific candidate genes (for detailed reviews see Razafsha et al., 2013; Renoir et al., 2013). In most cases, the modifications can only be applied in one direction, i.e., one can either apply a beneficial or an adverse treatment to the same model. Compared to that, the F1 g × e model provides the opportunity to modify environments beneficially or adversely. Further, as this model is based on a forward genetics approach leaving the genomes of the model organism intact and keeping the full spectrum of genetic and epigenetic contributors to our phenotypes of interest, it also provides the possibility to screen for candidate genes of environmental plasticity in an unbiased manner.

We could clearly demonstrate that total *Hmgn3* expression was altered depending on the environmental stimulus applied, thereby revealing a plasticity gene for environmental modifications. Although asGEX ratios were unaffected, the ratios were as expected from the parental lines, i.e., the different gene expression patterns in HAB *vs.* LAB mice is likely to be caused by the respective genetic sequence of these lines and, thus, inherited. For *Crhr1*, we did not observe different tGEX, but the environmental modifications shifted the ratios of asGEX. While this might have an impact on HAB *vs.* LAB divergence, it suggests that a functional contribution is less likely for this F1 model. Thus, the F1 g × e model provides the opportunity to study further environmental effects, including allele-specific methylation, as for instance Klengel et al. (2013) identified allele-specific methylation of FKBP5 as a potential mechanism mediating the development of posttraumatic stress disorder (PTSD).

*Hmgn3* (a member of the high mobility group N protein family) is known to regulate the transcription profile of eukaryotic cells by affecting the structure and function of chromatin and is strongly expressed in brain tissue (Kugler et al., 2013). This gene was found to be 2-fold higher expressed in HAB *vs.* LAB mice (Czibere et al., 2011). *Hmgn3* exhibited a similar pattern of asGEx in F1 hybrids as it was expected from the parental lines, assuming the expression ratio of roughly 2:1 from HAB:LAB. This indicates that the gene activity is highly influenced by functional polymorphisms, i.e., independent of transcription factors that differ between HAB and LAB mice. We observed significant differences of *Hmgn3* tGEx after EE or CMS. Thus, the bidirectional shift of trait anxiety is strongly correlated with *Hmgn3* tGEx. Irrespective of asGEx patterns that retained the proportions of basal expression in HAB *vs.* LAB, with a 1.1 kbp CpG island in the gene promoter (UCSC Genomer Browser), it could be regulated by methylation and thus makes *Hmgn3* susceptible to epigenetic modifications. Therefore, asGEx remains constant in reciprocal hybrids and the tGEx seems likely to be regulated by epigenetic modifications in this model, making *Hmgn3* a candidate gene of anxiety in a “gene × environment plasticity gene” construct in the HAB × LAB F1 intercross.

A dysregulated CRH/CRHR1 system is suggested to be one of the most common disturbances associated with psychiatric disorders (Arborelius et al., 1999; Müller et al., 2003; de Kloet et al., 2005; Ressler et al., 2010; Griebel and Holsboer, 2012) and critically involved in both the regulation of anxiety-related behavior and the reactivity of the hypothalamic-pituitary-adrenal axis (Reul and Holsboer, 2002). Recently, it has been shown that the expression of *Crhr1* in the amygdala is crucial for the anxiety state (Sztainberg et al., 2010; Rogers et al., 2013). Here we have shown that, although there is higher tGEx of *Crhr1* mRNA in the BLA of HAB in comparison to LAB mice, the asGEx in F1 mice did not retain the proportions observed in the parental mouse lines. This suggests an essential role of transcription factors in the regulation of tGEx in the parental lines, as it was highlighted for Yin-Yang 1 (Sotnikov et al., 2014). Our results point to this conclusion for *Crhr1* in an allele*-*specific manner: decrease in anxiety of F1 animals after EE is accompanied by a corresponding decrease in HAB asGEx and increase in LAB asGEx in the BLA. At the same time, CMS acting in the juxtaposed direction induced higher anxiety levels, accompanied by an increase in the HAB asGEx and a decrease in the amount of LAB allelic mRNA. However, no change in tGEx was observed upon treatment, therefore any phenotypic effect of *Crhr1* in this model might rather be due to different effects of the HAB- and LAB-specific alleles described here. Finally, we can only speculate, which cell types might be involved. Since a recent paper by Refojo et al. (2011) suggests that CRHR1 expression could mediate anxiogenic effects on glutamatergic neurons, and anxiolytic effects on dopaminergic neurons, the observed changes after EE and CMS are likely to involve the regulation of *Crhr1* particularly in these neurons.

Differences in the tGEx and asGEx could arise from a variety of sources: dissimilar ability of activating/suppressing transcription factors to bind to the promoters due to SNPs (Murata et al., 2012), allele-specific methylation of regulatory regions (Klengel et al., 2013), differences in mRNA stability (Shabalina et al., 2004), the haplotype structure of regulatory polymorphisms (Hudson, 2003) or gene copy number variations. Using four inbred mouse strains, Cowles et al. (2002) found that asGEx can also highly depend on tissue and environmental conditions.

Taken together, our data suggest that the bidirectional shift in phenotype by environmental modifications is strongly and stably correlated with a change in the asGEx of *Crhr1*, which strengthens our previous findings in environmental modifications in HAB and LAB mice. The reported change in tGEx of *Hmgn3* upon EE and UCMS further extends our documented plasticity genes of gene-environmental interactions in the regulation of anxiety-related traits.

## Author contributions

Natalia Yurievna Chekmareva, Sergey V. Sotnikov, Rebekka P. Diepold, Roshan R. Naik, Rainer Landgraf and Ludwig Czibere designed the experiments and wrote the manuscript, Natalia Yurievna Chekmareva, Sergey V. Sotnikov, Rebekka P. Diepold and Roshan R. Naik performed the experiments and analyzed the data with Ludwig Czibere.

## Conflict of interest statement

The authors declare that the research was conducted in the absence of any commercial or financial relationships that could be construed as a potential conflict of interest.
